# Connectome-Based Predictive Modeling of PTSD Development Among Recent Trauma Survivors

**DOI:** 10.1001/jamanetworkopen.2025.0331

**Published:** 2025-03-10

**Authors:** Ziv Ben-Zion, Alexander J. Simon, Matthew Rosenblatt, Nachshon Korem, Or Duek, Israel Liberzon, Arieh Y. Shalev, Talma Hendler, Ifat Levy, Ilan Harpaz-Rotem, Dustin Scheinost

**Affiliations:** 1 Department of Comparative Medicine, Yale University School of Medicine, New Haven, Connecticut; 2 Department of Psychiatry, Yale University School of Medicine, New Haven, Connecticut; 3 Clinical Neuroscience Division, Department of Veterans Affairs (VA) National Center for PTSD, VA Connecticut Healthcare System, West Haven; 4 School of Public Health, Faculty of Social Welfare and Health Sciences, University of Haifa, Haifa, Israel; 5 Department of Radiology, Yale School of Medicine, New Haven, Connecticut; 6 Department of Biomedical Imaging, Yale School of Medicine, New Haven, Connecticut; 7 Department of Epidemiology, Biostatistics, and Community Health Sciences, Ben-Gurion University of the Negev, Be’er-Sheva, Israel; 8 Department of Psychiatry, College of Medicine, Texas A&M, College Station; 9 Department of Psychiatry, NYU Grossman School of Medicine, New York, New York; 10 Sagol Brain Institute Tel Aviv, Wohl Institute for Advanced Imaging, Tel Aviv Sourasky Medical Center, Tel Aviv, Israel; 11 Sagol School of Neuroscience, Faculty of Social Sciences and Sackler Faculty of Medicine, Tel Aviv University, Tel Aviv, Israel; 12 Wu Tsai Institute, Yale University, New Haven, Connecticut; 13 Department of Psychology, Yale University, New Haven, Connecticut

## Abstract

**Importance:**

The weak link between subjective symptom-based diagnostics for posttraumatic psychopathology and objective neurobiological indices hinders the development of effective personalized treatments.

**Objective:**

To identify early neural networks associated with posttraumatic stress disorder (PTSD) development among recent trauma survivors.

**Design, Setting, and Participants:**

This prognostic study used data from the Neurobehavioral Moderators of Posttraumatic Disease Trajectories (NMPTDT) large-scale longitudinal neuroimaging dataset of recent trauma survivors. The NMPTDT study was conducted from January 20, 2015, to March 11, 2020, and included adult civilians who were admitted to a general hospital emergency department in Israel and screened for early PTSD symptoms indicative of chronic PTSD risk. Enrolled participants completed comprehensive clinical assessments and functional magnetic resonance imaging (fMRI) scans at 1, 6, and 14 months post trauma. Data were analyzed from September 2023 to March 2024.

**Exposure:**

Traumatic events included motor vehicle incidents, physical assaults, robberies, hostilities, electric shocks, fires, drownings, work accidents, terror attacks, or large-scale disasters.

**Main Outcomes and Measures:**

Connectome-based predictive modeling (CPM), a whole-brain machine learning approach, was applied to resting-state and task-based fMRI data collected at 1 month post trauma. The primary outcome measure was PTSD symptom severity across the 3 time points, assessed with the Clinician-Administered PTSD Scale for *DSM-5* (CAPS-5). Secondary outcomes included *Diagnostic and Statistical Manual of Mental Disorders* (Fifth Edition) (*DSM-5*) PTSD symptom clusters (intrusion, avoidance, negative alterations in mood and cognition, hyperarousal).

**Results:**

A total of 162 recent trauma survivors (mean [SD] age, 33.9 [11.5] years; 80 women [49.4%] and 82 men [50.6%]) were included at 1 month post trauma. Follow-up assessments were completed by 136 survivors (84.0%) at 6 months and by 133 survivors (82.1%) at 14 months post trauma. Among the 162 recent trauma survivors, CPM significantly predicted PTSD severity at 1 month (ρ = 0.18, *P* < .001) and 14 months (ρ = 0.24, *P* < .001) post trauma, but not at 6 months post trauma (ρ = 0.03, *P* = .39). The most predictive edges at 1 month included connections within and between the anterior default mode, motor sensory, and salience networks. These networks, with the additional contribution of the central executive and visual networks, were predictive of symptoms at 14 months. CPM predicted avoidance and negative alterations in mood and cognition at 1 month, but it predicted intrusion and hyperarousal symptoms at 14 months.

**Conclusions and Relevance:**

In this prognostic study of recent trauma survivors, individual differences in large-scale neural networks shortly after trauma were associated with variability in PTSD symptom trajectories over the first year following trauma exposure. These findings suggest that CPM may identify potential targets for interventions.

## Key Points

**Question:**

Can early functional connectivity within and between large-scale neural networks predict the development of posttraumatic stress disorder (PTSD) in recent trauma survivors?

**Findings:**

In this prognostic study of 162 adult trauma survivors, connectome-based predictive modeling applied to functional magnetic resonance imaging data at 1 month post trauma significantly predicted PTSD symptom severity at both 1 month and 14 months post trauma (but not at 6 months). Key predictive connections involved the anterior default mode, motor sensory, salience, central executive, and visual networks.

**Meaning:**

These findings suggest that early identification of neural network differences may guide targeted interventions to mitigate PTSD risk following trauma exposure.

## Teaser

This prognostic study uses connectome-based predictive modeling to identify early neural networks associated with posttraumatic stress disorder (PTSD) development among recent trauma survivors.

## Introduction

Posttraumatic stress disorder (PTSD) is a common and severe psychiatric condition with substantial public health implications due to its high prevalence, chronic nature, functional impairment, and frequent comorbidities.^1,2^ Longitudinal studies of the first year following trauma exposure reveal a spectrum of symptom trajectories, including low (64.5%), remitting (16.9%), moderate (6.7%), high (6.5%), and delayed (5.5%) symptoms.^3^ This variation in symptom trajectories, along with the persistence of nonremitting PTSD in some individuals, suggests long-lasting neurobehavioral modifications.^4^ Despite nearly 4 decades of research yielding substantial insights into the disorder’s underlying neurobiology, we still lack strong neural predictors of PTSD development or recovery, limiting the development of mechanism-based effective treatments. Currently, first-line therapeutics for PTSD—namely, pharmacotherapy and psychotherapy—show only limited efficacy in some individuals.^5,6^

Classical models of PTSD neural circuitry, derived mainly from univariate and bivariate analyses, highlight the role of the amygdala, hippocampus, and (medial) prefrontal cortex in the canonical “fear circuit” associated with the disorder’s symptoms.^7,8^ However, investigations have adopted network-based approaches (eg, independent component analysis, graph theory methods) and revealed disruptions in neural networks subserving salience, central executive, and default mode functions in PTSD.^9,10,11^ However, most studies are limited by their use of cross-sectional designs and focus on chronic PTSD populations, highlighting the need for longitudinal investigations in the early aftermath of trauma.^12,13^ Indeed, since 2020, there has been an increase in the number of neuroimaging studies conducted during the first year after trauma,^14,15,16^ a critical period for determining who will develop PTSD and who will recover.^17^

Longitudinal investigations of the multimodal dimensions of posttraumatic responses (eg, symptoms, cognitive functioning, and brain structure and function) during the first year post trauma are optimally suited to detect the underlying neurobehavioral moderators of PTSD development.^2,13,18^ Nevertheless, such studies are labor intensive and pose substantial technical and conceptual challenges. These challenges include reaching out to, enrolling, evaluating, and retaining participants in sufficient numbers to increase statistical power and decrease retention bias; determining the optimal timing of assessments, capturing both early acute responses and sufficient follow-up duration; minimizing participant burden and attrition, while still maintaining their engagement throughout; and choosing adequate methods and study designs to capture critical stages in PTSD development in a sensitive clinical population.^19,20^

From a methodologic point of view, machine learning approaches are increasingly used in research on trauma-related disorders, aiming to improve the classification of posttraumatic stress responses, predict clinical outcomes, or determine individual-specific treatment.^21,22^ Such approaches aim to reduce overfitting by being developed on a subset of the data (training set) and tested on the remainder of the data (test set), increasing the generalizability of their findings.^23,24^ One well-established machine learning approach is connectome-based predictive modeling (CPM), which generates predictive models linking brain features with specific phenotypes using whole-brain functional connectivity data (connectomes).^25^ This data-driven method requires no a priori selection of networks and thus can identify neural signatures of functional connectivity subserving a specific behavioral phenotype. Studies have applied CPM to identify neural signatures of several phenotypic measures, such as intelligence,^26^ attention,^27,28^ creativity,^29^ and craving.^30^ CPM has also successfully predicted clinical symptoms and treatment outcomes in a variety of neuropsychiatric disorders, including Alzheimer disease,^31^ attention-deficit/hyperactivity disorder,^27,28^ and substance use disorders.^30,32,33^ To date, only one study used CPM to predict PTSD severity in trauma survivors, highlighting the resting-state functional connectivity among the visual cortex, subcortical cerebellum, limbic, and motor systems.^34^ Nevertheless, the previous study was limited by its cross-sectional design and use of a single functional magnetic resonance imaging (fMRI) modality (resting state).^34^

To complement the existing literature, our work aimed to identify neural networks associated with PTSD development or recovery in a large-scale longitudinal neuroimaging dataset of recent trauma survivors.^13^ To do so, we applied CPM to functional brain connectivity shortly after trauma to predict PTSD symptom severity at several time points during the first critical year post trauma. First, we used both resting-state and task-based fMRI data of trauma survivors at 1 month post trauma to predict their PTSD symptom severity at 1, 6, and 14 months post trauma. At each time point, we further examined which specific brain networks contributed to the prediction of total PTSD severity and distinct PTSD symptom clusters. Virtual lesion analyses were used to test the specific networks that contributed to the prediction of PTSD severity. Based on prior neuroimaging research,^9,10,11^ we hypothesized that PTSD symptom severity would be associated with connectivity patterns within and among the salience network (SAL), default mode network (DMN), and central executive network (CEN) (ie, the triple-network model^35^).

## Methods

This prognostic study used data from a large-scale longitudinal neuroimaging dataset of recent trauma survivors collected between January 20, 2015, and March 11, 2020, as part of the Neurobehavioral Moderators of Posttraumatic Disease Trajectories (NMPTDT) study (ClinicalTrials.gov NCT03756545).^13^ The study was approved by the Tel Aviv Sourasky Medical Center Ethics Committee. All participants provided written informed consent in accordance with the Declaration of Helsinki^36^ and received financial remuneration of ₪800 (approximately US $220) at each assessment (1, 6, and 14 months post trauma). The NMPTDT study design and methods are detailed elsewhere,^13^ with key aspects relevant to this work summarized next. This study followed the Transparent Reporting of a Multivariable Prediction Model for Individual Prognosis or Diagnosis (TRIPOD) reporting guideline.

### Participants

Potential participants were civilians aged 18 to 65 years who were admitted to Tel Aviv Sourasky Medical Center’s emergency department (ED) after experiencing a traumatic event (eg, motor vehicle incident, bicycle incident, physical assault, robbery, hostilities, electric shock, fire, drowning, work accident, terror attack, or large-scale disaster). Individuals with any of the following were excluded: head injuries, unconsciousness upon ED admission, inability to consent, MRI-incompatible conditions (eg, pacemaker, metal implants), current substance use disorder, suicidal ideation, prior PTSD diagnosis, or history of psychotic disorder.

### Clinical Assessments

Trained and certified clinical interviewers assessed PTSD severity using the Clinician-Administered PTSD Scale for *DSM-5* (CAPS-5),^37^ comprising 20 items rated from 0 (absent) to 4 (extreme or incapacitating). The primary outcome measure (CAPS-5 total scores) was the sum of all symptom ratings (range, 0-80), with higher scores indicating greater PTSD severity. The secondary outcome measure was the total scores of the 4 *Diagnostic and Statistical Manual of Mental Disorders* (Fifth Edition) (*DSM-5*) PTSD symptom clusters: intrusion or reexperiencing (criterion B), avoidance (criterion C), negative alterations in mood and cognition (criterion D), and hyperarousal (criterion E).^38^

### Neuroimaging Data Acquisition and Preprocessing

Whole-brain functional and anatomical images were acquired on a 3.0-T MRI system (Siemens). At 1 month post trauma, each participant underwent 3 fMRI scans to measure brain activity during (1) a resting-state scan; (2) a face-matching task^39^ probing emotional reactivity and regulation; and (3) the Safe or Risky Domino Choice (SRDC) task^40^ assessing sensitivity to risk, punishment, and reward (eMethods in Supplement 1). The tasks were chosen to probe specific neurocognitive processes relevant to PTSD.

Preprocessing was conducted using fMRIPrep, version 1.5.8,^41^ as detailed in the eMethods in Supplement 1. Importantly, task-based functional connectivity was calculated from the raw time courses, without modeling specific blocks or conditions and without regressing out task-evoked activity. This approach leverages task-induced brain state manipulations, which have been shown to improve predictions of brain-behavior relationships.^42,43^

### Functional Connectivity Analysis

Preprocessed fMRI data were parcellated into 268 nodes using a whole-brain atlas,^44^ following protocols from previous CPM work.^26,27,28^ Functional connectomes were generated by correlating the blood oxygenation level–dependent time series between all node pairs, with each edge Fisher transformed. Connectomes of valid fMRI data for each participant were then averaged together (ie, all individuals in the final sample). This averaging increased the signal-to-noise ratio and demonstrated that the identified predictive connections were generalizable across different fMRI modalities (eg, resting-state and task-based fMRI), thus improving the accuracy of predictive modeling and contributing to better characterization of brain-behavior associations.^45^

### Procedure

**Figure 1.**
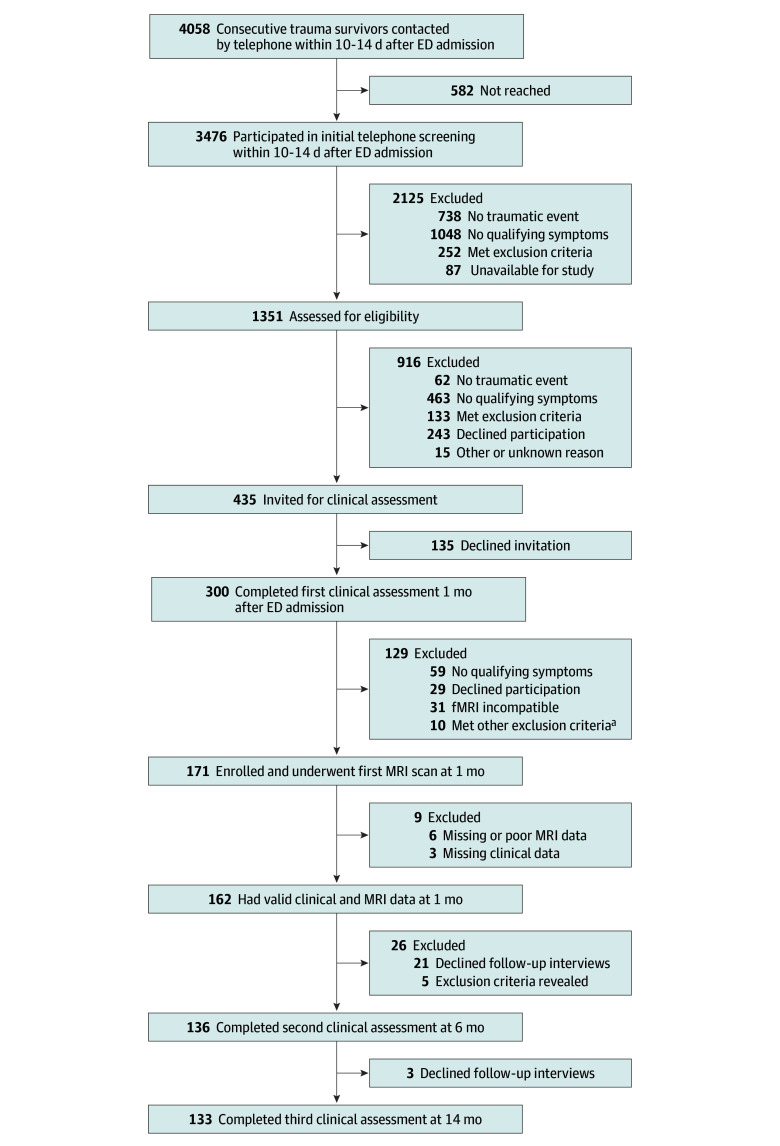
Study Flow Diagram ED indicates emergency department; fMRI, functional magnetic resonance imaging; MRI, magnetic resonance imaging. ^a^Other exclusion criteria were serious medical condition requiring clinical attention (n = 5), chronic posttraumatic stress disorder before the current event (n = 2), current substance use disorder (n = 1), head injury (n = 1), and no traumatic event (n = 1).

A total of 4058 consecutive trauma survivors admitted to the ED were contacted by telephone within 10 to 14 days after trauma exposure, were given information about the study, and provided informed assent (Figure 1). Of those, 3476 individuals were screened for psychological trauma and related symptoms, and 1351 were assessed for acute stress symptoms (indicative of chronic PTSD risk^46^). Among 435 eligible individuals invited for in-person clinical interviews, 300 attended; 171 individuals with qualifying symptoms underwent fMRI assessments within 1 month post trauma. Nine participants were excluded from this study due to missing MRI data (n = 6) or clinical data (n = 3), yielding a final sample of 162 participants at 1 month post trauma (eTable 1 in Supplement 1). Follow-up clinical assessments were completed by 136 participants at 6 months post trauma and 133 participants at 14 months post trauma.

### Statistical Analysis

CPM and virtual lesion analysis were performed as described next. Data were analyzed from September 2023 to March 2024.

#### Connectome-Based Prediction Modeling

Analysis was conducted using previously validated custom scripts for MATLAB release R2023b, version 23.2 (MathWorks).^25^ Edges positively and negatively associated with CAPS-5 total scores were selected using a threshold of *P* < .05, controlling for potential confounds (eg, head motion, age, and sex) through partial correlation. We used a 10-fold cross-validation with 100 random repeated divisions, using the Spearman rank correlation to assess model performance. Significance testing involved generating null distributions by randomly shuffling the correspondence between behavioral variables and connectivity matrices 1000 times, performing a CPM analysis for each shuffle. As in prior work,^26,27,28,32^ *P* values for predictions were calculated based on these null distributions. Because only a positive association between predicted and actual values can indicate prediction above chance (with negative associations indicating a failure to predict), 1-tailed *P* values are reported.

Our primary analysis used 1-month posttrauma neuroimaging data (resting-state, face-matching, and SRDC tasks) to predict PTSD severity (CAPS-5 total scores) at 1, 6, and 14 months following trauma exposure. Exploratory analyses assessed whether specific *DSM-5* PTSD symptom clusters (criteria B, C, D, and E) influenced PTSD severity predictions, using separate models trained for each cluster with *P* values corrected for multiple comparisons via false discovery rate (FDR).^47^ All analyses controlled for participant age and sex, as well as initial PTSD severity (CAPS-5 total scores at 1 month) for follow-up data (6 and 14 months post trauma).

#### Virtual Lesion Analysis

To determine the contribution of each network to prediction performance at 1, 6, and 14 months post trauma, we performed virtual lesion analysis of the anterior DMN (aDMN), CEN, posterior DMN, motor sensory network (MSN), visual network 1, visual network 2, visual association network, SAL, subcortical network, and cerebellar network. Each network was sequentially isolated within the CPM model, with all others masked out. We ran separate models for each network where every edge was virtually lesioned out, except for those within and between the network of interest. Single networks that outperformed the whole-brain connectomes in predictions were identified as driving predictions.

## Results

This study included 162 recent trauma survivors (mean [SD] age, 33.9 [11.5] years; 80 women [49.4%] and 82 men [50.6%]) with valid clinical and neuroimaging data at 1 month post trauma (Figure 1). Participants were predominantly exposed to motor vehicle incidents (143 [88.3%]) (eTable 1 in Supplement 1 presents demographic and clinical characteristics). Follow-up clinical assessments were completed by 136 participants (84.0%) at 6 months and by 133 participants (82.1%) at 14 months post trauma. No statistically significant differences in terms of age, sex distribution, or trauma type were observed between the 133 participants who completed all 3 assessments and the 29 participants (17.9%) lost to follow-up.

### Prediction of PTSD Symptom Development

**Figure 2.**
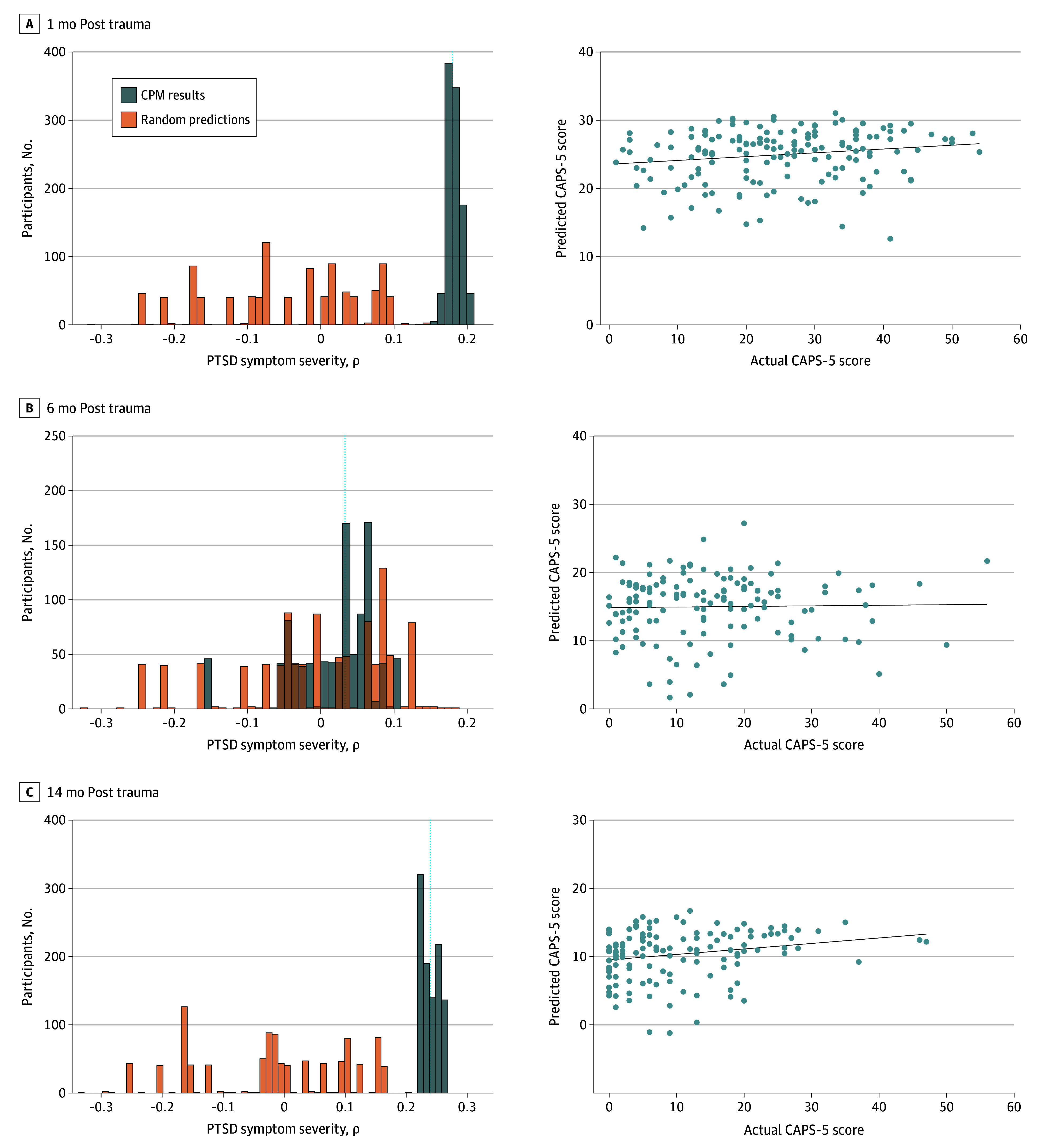
Prediction of Development of Posttraumatic Stress Disorder (PTSD) Symptoms A to C, Correlations between connectome-based predictive modeling (CPM)–predicted Clinician-Administered PTSD Scale for *DSM-5* (CAPS-5) total scores and actual total scores at 1 month (A), 6 months (B), and 14 months (C) post trauma. Left panels: For each time point, dashed lines mark the median of these correlations, and bars indicate CPM predictions and null distributions. Right panels: For each time point, scatter plots present the correlations between CPM-predicted and actual CAPS-5 total scores.

A 10-fold CPM trained and tested in the dataset was able to significantly predict PTSD symptom severity (ie, CAPS-5 total scores) at both 1 month (ρ = 0.18, *P* < .001) and 14 months (ρ = 0.24, *P* < .001) post trauma (Figure 2). The prediction of CAPS-5 total scores was not significant at 6 months post trauma (ρ = 0.03, *P* = .39). Numerically, predictions were greatest at the time point furthest away from the trauma (ie, 14 months), potentially highlighting the utility of CPM for predicting symptom trajectory. Given that different fMRI tasks (ie, resting-state, face-matching, and SRDC tasks) were combined in the prediction model, we further investigated the unique prediction of each scan. Overall, the connectomes from the resting-state and face-matching tasks performed better than those from the SRDC task (eTable 2 in Supplement 1).

### Brain Networks and Symptom Predictions

**Figure 3.**
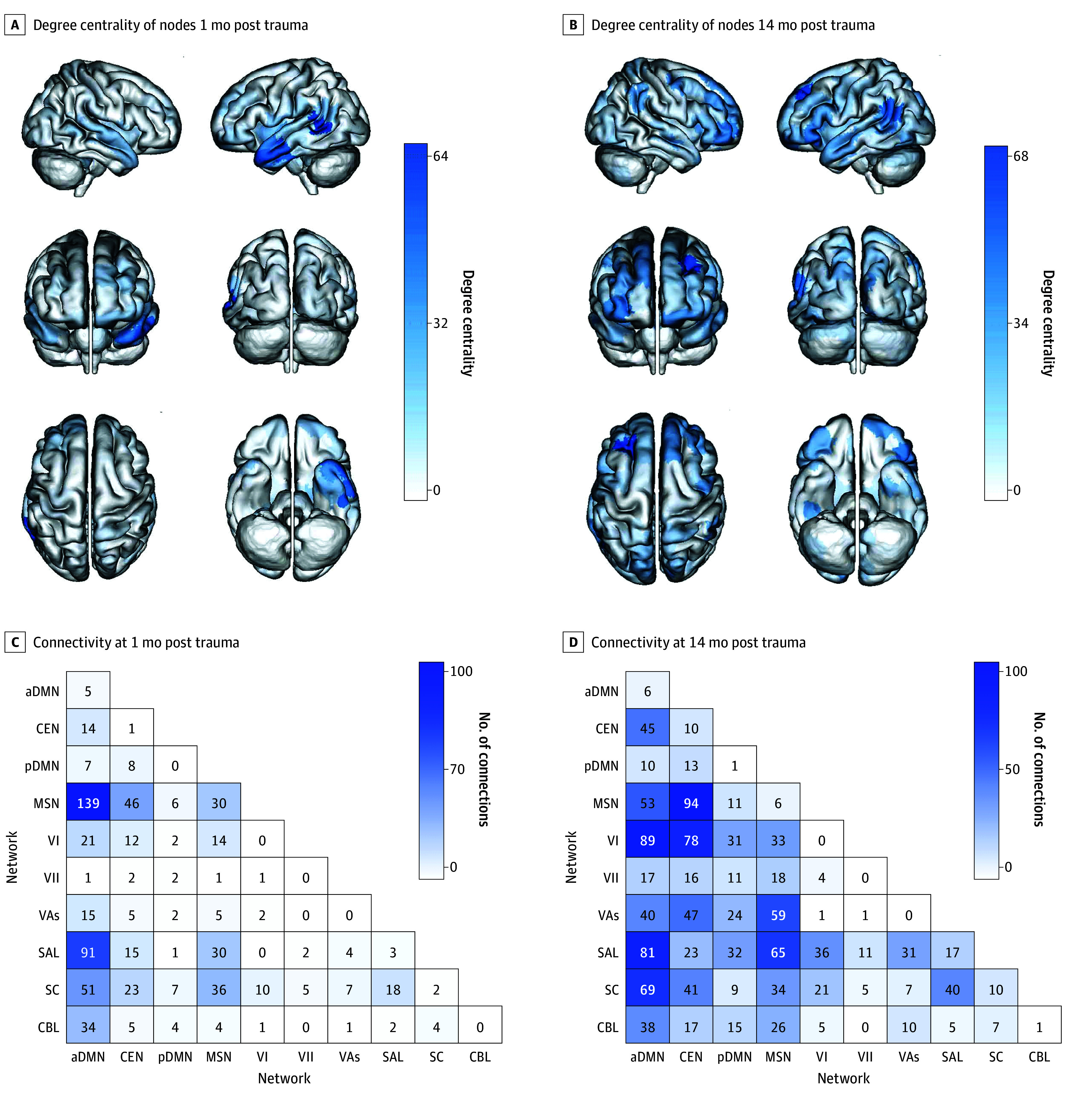
Brain Networks Contribute to Symptom Predictions Node-level and network-level contributions to predicting posttraumatic stress disorder (PTSD) symptom severity across time. Because only a few edges were positively associated with Clinician-Administered PTSD Scale for *DSM-5* (CAPS-5) total scores (as described in the Results), we present here only edges negatively associated with CAPS-5 total scores (ie, decreased connectivity associated with increased PTSD symptom severity). A and B, Degree centrality of nodes that negatively predicted CAPS-5 scores at 1 month (A) and 14 months (B) post trauma. Darker colors represent increased network centrality. C and D, Connections within and between canonical functional networks at 1 month (C) and 14 months (D) post trauma. The diagonal represents the average contribution of edges within a single network, and off-diagonal elements represent the average contribution of edges between 2 network pairs. Darker colors represent networks that contributed more toward the final prediction. aDMN indicates anterior default mode network; CBL, cerebellar network; CEN, central executive network; MSN, motor sensory network; pDMN, posterior default mode network; SAL, salience network; SC, subcortical network; VAs, visual association network; VI, visual network 1; VII, visual network 2.

**Figure 4.**
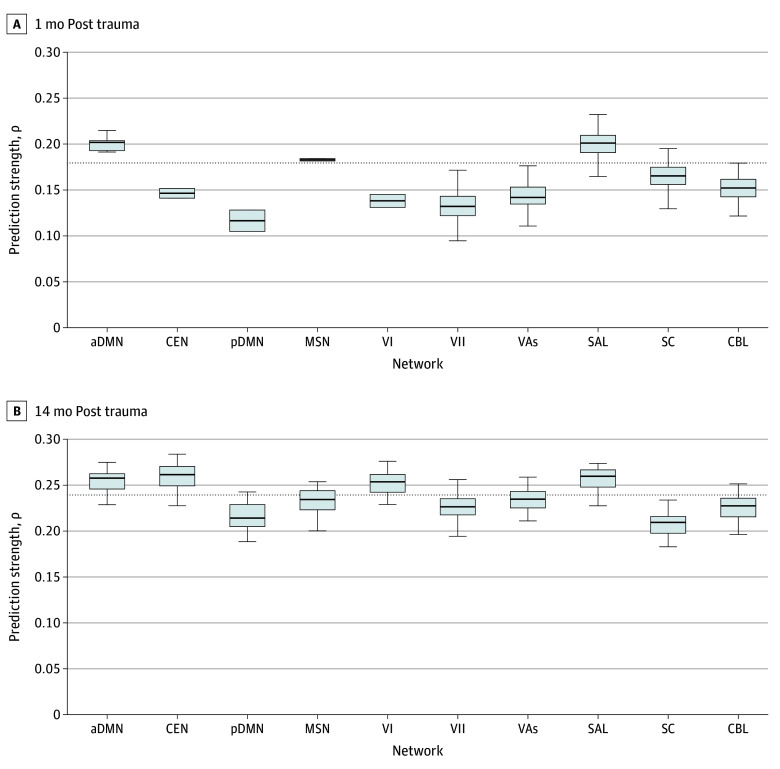
Lesion Analysis Results A and B, Spearman rank correlations between the predicted symptom severity and actual symptom severity (Clinician-Administered PTSD Scale for *DSM-5* total scores) from virtual lesion analyses at 1 month (A) and 14 months (B) post trauma. The whole-brain predictions at each time point are indicated by the horizontal dotted lines. Boxes indicate the IQR, and the solid lines within the boxes indicate the median; whiskers indicate the minimum and maximum values within 1.5 times the IQR. Single canonical networks that predicted better than the whole-brain connectomes were identified as driving predictions. aDMN indicates anterior default mode network; CBL, cerebellar network; CEN, central executive network; MSN, motor sensory network; pDMN, posterior default mode network; SAL, salience network; SC, subcortical network; VAs, visual association network; VI, visual network 1; VII, visual network 2.

Next, we examined the localization of the brain networks that contributed to the statistically significant PTSD symptom predictions (Figure 3). At 1 month post trauma, there were a total of 715 predictive edges. Only 14 edges (2.0%) positively predicted PTSD symptoms (ie, greater connectivity predicted more severe symptoms), whereas 701 (98.0%) negatively predicted symptoms (ie, decreased connectivity predicted more severe symptoms). Most negatively predictive edges were connected to the aDMN and its connectivity with the MSN and the SAL (Figure 3C). Indeed, results of the virtual lesion analysis suggested that the aDMN, MSN, and SAL were the most important for prediction, because they outperformed whole-brain CPM models at 1 month post trauma (Figure 4 and eTable 3 in Supplement 1).

At 14 months post trauma, there were a total of 1377 predictive edges. Only 3 edges (0.2%) positively predicted PTSD symptoms, whereas 1374 (99.8%) negatively predicted symptoms. Compared with the 1-month prediction model, negatively predictive edges were more broadly distributed over a larger number of brain networks (Figure 3D). This included connections between and within the aDMN, CEN, SAL, MSN, and visual networks. Indeed, virtual lesion analysis suggested that the aDMN, CEN, SAL, and visual networks outperformed the whole-brain CPM models at 14 months post trauma (Figure 4 and eTable 3 in Supplement 1). The MSN and visual association network performed similar to the whole-brain models.

Unique and shared edges from the 1-month and 14-month posttrauma models are presented in eFigures 1 and 2 in Supplement 1. The specific nodes that contributed to predicting PTSD symptoms at each time point are presented in the eResults and eTable 4 in Supplement 1.

### Prediction of Specific PTSD Symptom Clusters

Consistent with CAPS-5 total score predictions, CPM was able to significantly predict specific *DSM-5* PTSD symptom clusters at 1 month and 14 months post trauma, but not at 6 months post trauma (Table). At 1 month post trauma, the predictions were significant for avoidance symptoms (criterion C: ρ = 0.18, FDR-corrected *P* = .003) and negative alterations in mood and cognition (criterion D: ρ = 0.18, FDR-corrected *P* = .003), after correcting for multiple comparisons. However, they did not significantly predict intrusion symptoms (criterion B) or hyperarousal symptoms (criterion E) (Table). An opposite pattern was observed at 14 months post trauma, in which significant predictions were observed for intrusion symptoms (ρ = 0.22, FDR-corrected *P* = .003) and hyperarousal symptoms (ρ = 0.26, FDR-corrected *P* = .003), but not for avoidance or negative alterations in mood and cognition (Table). Details on the localization of the predictive edges for each symptom cluster at 1 month and 14 months are presented in eFigures 3 and 4 in Supplement 1.

**Table.** Correlations Between Predicted and Actual Symptom Severity Scores for Each Symptom Cluster During Posttrauma Assessment

PTSD symptom cluster (*DSM-5* criterion)	Posttrauma assessment period					
	1 mo		6 mo		14 mo	
	ρ	*P* value^a^	ρ	*P* value	ρ	*P* value
Intrusion or reexperiencing (B)	0.12	.12	0.04	.35	0.22	.003
Avoidance (C)	0.18	.003	0.10	.24	0.08	.24
Negative alterations in mood and cognition (D)	0.18	.003	−0.01	.38	0.16	.24
Hyperarousal (E)	0.16	.12	0.05	.47	0.26	.003

Abbreviations: *DSM-5*, *Diagnostic and Statistical Manual of Mental Disorders* (Fifth Edition); PTSD, posttraumatic stress disorder.

^a^ *P* values were false discovery rate corrected.

## Discussion

In this study, we used CPM to pinpoint early neural networks associated with PTSD development and recovery, both at rest and during tasks. Using data from a large-scale longitudinal neuroimaging dataset of recent trauma survivors,^13^ we observed that decreased functional connectivity at 1 month post trauma—primarily within and between the aDMN, SAL, and MSN—was associated with more severe PTSD symptoms. A broader pattern of decreased functional connectivity at 1 month, involving the CEN and visual networks in addition to the previous ones, was predictive of more severe symptoms at 14 months post trauma. Furthermore, our CPM model predicted different *DSM-5* PTSD symptom clusters at 1 month (avoidance and negative alterations in mood and cognition) compared with 14 months post trauma (intrusion and hyperarousal). These findings highlight that individual differences in early functional connectivity of neural networks contribute to divergent PTSD symptom trajectories during the first critical year post trauma, presenting opportunities for targeted intervention strategies.

Consistent with the CPM approach, we identified complex and distributed neural networks at 1 month post trauma that predicted PTSD development (or recovery) over the subsequent 14 months. As hypothesized, altered connectivity within and among the SAL, aDMN, and CEN correlated with PTSD severity, aligning with the triple-network model of the disorder.^10,48,49,50^ Notably, the first 2 networks (SAL, DMN) were linked to PTSD severity at both 1 month and 14 months post trauma, whereas the CEN only showed predictive value at 14 months, potentially reflecting deficits in executive functioning as the disorder progresses. Additionally, our findings highlighted the involvement of the visual networks and the MSN, echoing results from the only previous CPM study of PTSD in trauma survivors.^34^ More broadly, PTSD is associated with altered sensory processing,^51^ especially visual processing.^52^ For example, severe emotional trauma can trigger persistent and intense unpleasant sensory memories that disrupt visual processing already at the preattentive level.^53^ In a 2020 study, researchers further demonstrated that increased functional connectivity between somatosensory and visual networks following trauma-focused psychotherapy was associated with better treatment outcomes.^54^

In this study, the CPM model, derived from whole-brain functional connectivity at 1 month post trauma, did not predict PTSD symptom severity at 6 months post trauma. This finding might reflect the disorder’s dynamic nature and the substantial variability in clinical symptoms among individuals in the first year post trauma.^55^ The 6-month mark may be too early to indicate a chronic form of PTSD,^56^ which typically becomes distinguishable and stable by 14 months post trauma, with little likelihood of subsequent recovery.^17^ This finding is consistent with previous research that failed to identify early neuroimaging predictors of PTSD severity at 6 months post trauma.^40,57,58,59^

Although there is no definitive evidence that functional connectivity outperforms other fMRI metrics in predicting clinical outcomes,^60^ it offers benefits for brain-based models by capturing network architecture and organization, which are often disrupted in psychiatric disorders.^61^ This study integrated whole-brain functional connectivity from resting-state and task-based fMRI, 2 typically separate modalities, providing a comprehensive view of both intrinsic and task-evoked neural network architectures.^62^ Because large-scale longitudinal neuroimaging studies on posttraumatic stress response are rare (due to technical and conceptual challenges^58,63,64^), the use of the NMPTDT dataset^13^ for CPM development and validation marks a notable strength of this study. Finally, by focusing on PTSD symptom severity rather than a binary diagnosis of the disorder, this study provides a comprehensive view of brain-behavior relationships in the early aftermath of trauma.

### Limitations

This study has several limitations. First, although the CPM model identified altered connectivity of the medial prefrontal cortex (aDMN), it did not detect abnormal connectivity of the amygdala or hippocampus, key areas of the fear circuit linked to PTSD symptoms.^7,8,65,66^ This result may be due to the relatively small sizes of these regions compared with other nodes in the 268-node functional atlas,^67,68^ as well as to the fact that CPM is data driven and not ideally suited for testing specific region of interest–based hypotheses.^69^ Nevertheless, our results highlight the importance of widespread functional brain networks in predicting PTSD symptoms. Second, we focused on functional connectivity as a primary measure, but incorporating other imaging metrics (eg, activation patterns, cortical and subcortical volumes, or diffusion measures) could provide additional insights into the multifaceted nature of PTSD. Third, because the NMPTDT sample included individuals who experienced traumatic events mostly in the form of motor vehicle incidents (143 of 162 [88.3%]), these results require further replications in samples with other trauma types^70,71^ (eg, terror attacks, sexual assaults). Finally, although we used *DSM-5–*based classification of PTSD symptom clusters (criteria B, C, D, and E), future studies could benefit from testing whole-brain functional connectivity against an alternative, theory-driven classification of PTSD^72^ to address the limitations of the traditional diagnostic systems.^5,73^ Using alternative classifications of PTSD may also improve the observed modest effect sizes.^74^

## Conclusions

This prognostic study used CPM to identify individual differences in functional connectivity patterns within large-scale neural networks, shortly after trauma. These findings suggest that CPM may assist in the objective prediction of divergent PTSD symptom trajectories in the first 14 months post trauma and enhance our understanding of PTSD development and recovery, opening new avenues for personalized treatment strategies tailored to the underlying neural mechanisms.

## Notes

Supplement 1.
eMethods

eResults
**eTable 1.** Participant Demographic and Clinical Characteristics **eTable 2.** Unique Predictions of Each Functional Magnetic Resonance Imaging Task **eTable 3.** Lesion Analyses Results **eTable 4.** Top Predicting Nodes **eFigure 1.** Unique Edges Predicting PTSD Symptoms at 1 Month vs 14 Months **eFigure 2.** Shared Edges Predicting PTSD Symptoms at Both 1 Month and 14 Months **eFigure 3.** Brain Networks Contributing to Specific PTSD Symptom Clusters at 1 Month **eFigure 4.** Brain Networks Contributing to Specific PTSD Symptom Clusters at 14 Months
eReferences

Supplement 2.
Data Sharing Statement
