# Supplementary material for: Connectome-Based Predictive Modeling of PTSD Development Among Recent Trauma Survivors
Source: JAMA Netw Open. 2025 Mar 10;8(3):e250331. doi: 10.1001/jamanetworkopen.2025.0331 (PMC11894499; doi:10.1001/jamanetworkopen.2025.0331)
Supplement: Supplement 1. — eMethods eResults eTable 1. Participant Demographic and Clinical Characteristics eTable 2. Unique Predictions of Each Functional Magnetic Resonance Imaging Task eTable 3. Lesion Analyses Results eTable 4. Top Predicting Nodes eFigure 1. Unique Edges Predicting PTSD Symptoms at 1 Month vs 14 Months eFigure 2. Shared Edges Predicting PTSD Symptoms at Both 1 Month and 14 Months eFigure 3. Brain Networks Contributing to Specific PTSD Symptom Clusters at 1 Month eFigure 4. Brain Networks Contributing to Specific PTSD Symptom Clusters at 14 Months eReferences [file jamanetwopen-e250331-s001.pdf]

Supplemental Online Content

Ben-Zion Z, Simon AJ, Rosenblatt M, et al. Connectome-based predictive modeling of PTSD development among recent trauma survivors. *JAMA Netw Open*. 2025;8(3):e250331. doi:10.1001/jamanetworkopen.2025.0331

eMethods

eResults

eTable 1. Participant Demographic and Clinical Characteristics

eTable 2. Unique Predictions of Each Functional Magnetic Resonance Imaging Task

eTable 3. Lesion Analyses Results

eTable 4. Top Predicting Nodes

eFigure 1. Unique Edges Predicting PTSD Symptoms at 1 Month vs 14 Months

eFigure 2. Shared Edges Predicting PTSD Symptoms at Both 1 Month and 14 Months

eFigure 3. Brain Networks Contributing to Specific PTSD Symptom Clusters at 1 Month

eFigure 4. Brain Networks Contributing to Specific PTSD Symptom Clusters at 14 Months

eReferences

This supplemental material has been provided by the authors to give readers additional information about their work.

eMethods

Neuroimaging Data Acquisition. Whole-brain functional and anatomical images were acquired with a 3.0-T Siemens MRI system (MAGNETOM Prisma, Germany) with a 20-channel head coil. Functional images were acquired in an interleaved order (anterior to posterior) with a T2*-weighted gradient-echo planar imaging pulse sequence (TR=2,000 ms, TE=28 ms, flip angle=90°, voxel size=2.2 mm^3, FOV=224*224 mm, slice thickness=3 mm, 36 slices per volume). A T1-weighted three-dimensional anatomical image was obtained with a magnetization-prepared rapid gradient echo (MPRAGE) sequence (TR=2,400 ms, TE=2.29 ms, flip angle=8°, voxel size=0.7 mm^3, FOV=224*224 mm) to enable optimal localization of the functional effects.

Three functional scans were utilized for connectome-based predictive modeling (CPM) of posttraumatic stress disorder (PTSD) symptoms. First, we used data from the resting-state scan (duration = 10 min, TR = 2000 ms, TE = 28 ms, voxel size=3mm^3), in which participants were instructed to keep their eyes open and fixed on a fixation cross. Second, we used data from the well-known face-matching task[1], which probes participants' reactivity to socially threatening cues. In this paradigm, individuals were instructed to select the face (located at the bottom right or bottom left of the screen) that matches the target face (located at the top of the screen), as accurately and as quickly as possible. The task included four blocks of shapes, and four blocks of emotional faces (angry, fearful, surprised, and neutral faces). The order of the emotional face blocks was counterbalanced between subjects, as well as within-subjects across different time points, using four different orders for this task. Third, we used data from the "Safe or Risky Domino Choice" (SRDC) task[2], an interactive naturalistic gambling game. The effectiveness of the SRDC in detecting individuals' sensitivity to risk, punishment, and reward was previously validated in both healthy and clinical populations[3–8]. In this task, individuals played a 2-player competitive game, in which they were required to make risky choices to win. Each round of the game is composed of four intervals: decision-making, decision-execution, anticipation of an outcome, and response to outcomes (for full details, see Ben-Zion et al. (2022)[2]). Importantly, we did not model the different blocks or conditions of the two tasks (i.e., face-matching task, SRDC task) for the CPM analysis, but used the whole BOLD time series as typically done with resting-state data.

fMRI Preprocessing. For the neuroimaging data preprocessing, raw DICOM data images were converted to NIFTI format and organized to conform to the 'Brain Imaging Data Structure' specifications (BIDS)[9] Quality control steps of both the anatomical and functional data were conducted using: (1) Visual inspection for conversion errors and data exclusion criteria (e.g., anatomical abnormalities, signal drop-out); (2) MRI Quality Control (MRIQC)[10] tool developed by the Poldrack Lab at Stanford University for use at the Center for Reproducible Neuroscience (CRN). Preprocessing was conducted using fMRIPrep version 1.5.8[11], a Nipype-based tool[12], and will be described separately for the anatomical and functional data.
(1) Anatomical data preprocessing. Within the fMRIPrep framework, T1-weighted (T1w) images were corrected for intensity non-uniformity (INU) using `N4BiasFieldCorrection'[13] version 2.1.0, distributed with `AntsApplyTransforms` (ANTs) version 2.2.0. The T1w-reference was then skull-stripped with a Nipype implementation of the `antsBrainExtraction.sh` workflow (ANTs version 2.2.0), using OASIS30-ANTs as a target template. A T1w-reference map was computed after registration of the INU-corrected T1w images using `mri_robust_template` (FreeSurfer version 6.0.1). Volume-based spatial normalization to the ICBM 152 Nonlinear Asymmetrical template version 2009c ('MNI152NLin2009cAsym')[14] was performed through nonlinear registration with `antsRegistration` tool (ANTs version 2.2.0), using brain-extracted versions of both T1w volume and template. Brain tissue segmentation of cerebrospinal fluid (CSF), white matter (WM), and gray matter (GM) was performed on the brain-extracted T1w images using FMRIB's Automated Segmentation Tool ('FAST'), as part of FSL version 5.0.9[15].
(2) Functional data preprocessing. For each of the BOLD runs found per subject (across all tasks and sessions), the following preprocessing was performed. First, a reference volume and its skull-stripped version were generated using a custom methodology of fMRIPrep. Susceptibility distortion correction (SDC) was omitted. The BOLD reference was then co-registered to the T1w reference using 'bbregister' (FreeSurfer) which implements boundary-based registration[16]. Co-registration was configured with 9 degrees of freedom to account for distortions remaining in the BOLD reference. Head-motion parameters for the BOLD reference (transformation matrices, and six corresponding rotation and translation parameters) were estimated before any spatiotemporal filtering using `mcflirt` (FSL version 5.0.9)[17]. BOLD runs were slice-time corrected using `3dTshift` from AFNI version 16.2.07. The BOLD time-series (including slice-timing correction) were resampled onto their original, native space by applying the transforms to correct for head motion. These resampled BOLD time-series will be referred to as 'preprocessed BOLD in original space', or just 'preprocessed BOLD'. First, a reference volume and its skull-stripped version were generated using a custom methodology of fMRIPrep. Several confounding time-series were calculated based on the

© 2025 Ben-Zion Z et al. *JAMA Network Open*.

preprocessed BOLD: framewise displacement (FD), DVARS, and three region-wise global signals (extracted within the CSF, WM, and whole-brain masks). Additionally, a set of physiological regressors was extracted to allow for component-based noise correction (‘CompCor’)[18]. Principal components were estimated after high-pass filtering of the pre-processed BOLD time-series (using a discrete cosine filter with 128s cut-off) for the two ‘CompCor’ variants: temporal (‘tCompCor’) and anatomical (‘aCompCor’). Six ‘tCompCor‘ components were then calculated including only the top 5% variable voxels within that subcortical mask. For ‘aCompCor’, six components were calculated within the intersection of the subcortical mask, and the union of CSF and WM masks was calculated in T1w space, after their projection to the native space of each functional run. For each CompCor decomposition, the k components with the largest singular values were retained, sufficient to explain 50% of the variance across the nuisance mask. The remaining components were dropped from consideration. The head-motion estimates calculated in the correction step were also placed within the corresponding confounds file. The confound time series derived from head motion estimates and global signals were expanded with the inclusion of temporal derivatives and quadratic terms. Frames that exceeded a threshold of 0.5mm FD or 1.5 standardized DVARS were annotated as motion outliers. All re-samplings were performed with a single interpolation step by composing all the pertinent transformations. Gridded (volumetric) re-samplings were performed using ‘antsApplyTransforms’ (ANTs), configured with Lanczos interpolation to minimize the smoothing effects of other kernels[19]. Non-gridded (surface) re-samplings were performed using `mri_vol2surf` (FreeSurfer). Many internal operations of FMRIPREP use `Nilearn` version 0.6.2[20], mostly within the BOLD-processing workflow. Finally, spatial smoothing was performed with an isotropic Gaussian kernel of 6mm FWHM (full-width at half-maximum).

Functional Connectivity Analysis. Nodes were defined using the Shen 268-node brain atlas, which includes the cortex, subcortex, and cerebellum as described in prior CPM work[21,22]. The atlas was warped from MNI space into single-subject space via a series of linear and non-linear transformations. Task connectivity was calculated based on the ‘raw’ task time courses, with no regression of task-evoked activity, which emphasizes individual differences in connectivity[23]. This involved computation of the mean time courses for each of the 268 nodes (i.e., averaging the time courses of all constituent voxels). Node-by-node pairwise correlations were computed, and Pearson correlation coefficients were Fisher z-transformed to yield symmetric 268x268 connectivity matrices, in which each matrix element represents the connectivity strength between two individual nodes (i.e., ‘edge’).
Regarding sample sizes for each scan, n=137 individuals had valid data for all three functional scans (rest, face matching task, and SRDC task). Furthermore, 21 participants had valid data for two (out of three) scans: rest and SRDC task (n=7), rest and face-matching task (n=12), or SRDC and face-matching tasks (n=2). Finally, 4 participants had only one type of scan, either rest (n=2), SRDC task (n=1), and face-matching task (n=1). In summary, n=158, n=146, and n=152 had valid data for the rest scan, SRDC task, and face-matching task, respectively. The averaging of scans resulted in a final sample size of 162 individuals.

Connectome-Based Prediction Modeling (CPM). During CPM, edges (correlations between nodes in functional connectivity matrix) and behavioral data (PTSD symptoms) from a training dataset are correlated using regression analyses to identify positive and negative predictive networks (networks not shown). Single-subject summary statistics are created as the sum of the edge weights in each network and used to create predictive models assuming linear relationships with behavioral data. Resultant polynomial coefficients are applied to the novel subject’s FC matrices (i.e., test data) to generate behavioral predictions. Model performance is evaluated based on the correlation between actual and predicted PTSD symptoms. Matlab scripts of the main CPM analyses can be found at https://github.com/asimon445/PTSD_CPM.

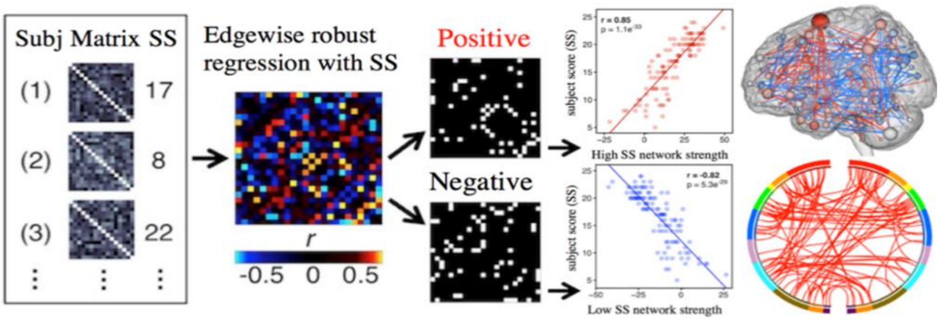

© 2025 Ben-Zion Z et al. *JAMA Network Open*.

eResults

Clinical PTSD symptoms across time. At 1-month post-trauma, CAPS-5 total scores ranged from 1 to 54, with a mean of 24.94 and standard deviation of 11.80. At 6-months, CAPS-5 total scores ranged from 0 to 56 (mean=15.08, SD=11.38), and at 14-months, scores ranged from 0 to 47 (mean=10.45, SD=10.06).

Brain regions contributing to symptom predictions. The top predicting nodes at 1-month and 14-months post-trauma are presented in eTable 4. Overall, the left dorsolateral PFC (dlPFC) was the most predictive region of PTSD symptoms at both time points. Other regions that contributed to the prediction at both 1-month and 14-months were pars orbitalis of the inferior frontal gyrus (IFG), angular gyrus, medial temporal gyrus, anterior PFC, supramarginal gyrus, frontal eye-fields (prefrontal) regions, and secondary visual (occipital) areas (see eTable 4). Notably, three regions were associated with symptoms only at 1-month post-trauma: left Broca-operculum, left temporal pole, and right pars orbitalis of the IFG. On the other hand, three regions were predictive of PTSD symptoms only at 14-months post-trauma: right pars orbitalis of the IFG, left anterior PFC, and right inferior temporal gyrus.

© 2025 Ben-Zion Z et al. *JAMA Network Open*.

eTable 1. Participant Demographic and Clinical Characteristics

MVA = Motor vehicle accident; PCL-4 = PTSD Checklist for DSM-IV; CAPS = Clinician-Administered PTSD Scale for DSM-IV (CAPS-4) or for DSM-5 (CAPS-5).

**Demographic & Clinical Characteristics (NMPTDT, n=162)**		
**Measure**	***M***	***SD***
Age (Years)	33.92	11.48
Education (Years)[a]	14.25	2.62
CAPS-5 (Total Score)	24.94	11.80
CAPS-4 (Total Score)	51.68	22.67
PCL-4 (Total Score)	46.79	14.32
**Measure**	**n [%]**	
Biological Sex	Female: 80 [49%]	
	Male: 82 [51%]	
Marital Status[b]	Single: 107 [69%]	
	Married: 32 [21%]:	
	Divorced: 15 [10%]	
Trauma Type	MVA: 143 [88%]	
	Assault: 11 [7%]	
	Other: 8 [5%]	

[a] n=11 participants did not report their education level (in years).
[b] n=5 participants did not provide information about their marital status.

© 2025 Ben-Zion Z et al. *JAMA Network Open*.

eTable 2. Unique Predictions of Each Functional Magnetic Resonance Imaging Task

Spearman's rank correlations between the predicted PTSD symptom severity scores and actual symptom severity scores from each task separately: Resting-state scan ("Resting"), Face-matching task ("Face-matching")[1], "Safe or Risky Domino Choice" (SRDC) task[2]. Results are presented separately for 1-month and 14-months post-trauma. The corresponding p-values are FDR-corrected for multiple comparisons. **pFDR < 0.001.

	**1-month**	**14-months**
**Resting-state**	*rho* = 0.1632<br>$p_{FDR} < 0.001$**	*rho* = 0.1843<br>$p_{FDR} < 0.001$**
**Face-matching task**	*rho* = 0.1403<br>$p_{FDR} < 0.001$**	*rho* = 0.2437<br>$p_{FDR} = 0.001$**
**SRDC task**	*rho* = 0.1331<br>$p_{FDR} = 0.086$	*rho* = 0.1509<br>$p_{FDR} = 0.051$

© 2025 Ben-Zion Z et al. *JAMA Network Open*.

eTable 3. Lesion Analyses Results

Spearman's rank correlations between the predicted symptom severity and actual symptom severity (CAPS-5 total scores) from virtual lesion analyses at 1- and 14-months post-trauma. Single canonical networks that predicted better than whole-brain connectomes at each time point were identified as driving predictions (yellow).

	**1-month lesions**	**14 months lesions**
**Anterior Default Mode Network (aDMN)**	rho = 0.2018<br>p_{FDR} = 0.001	rho = 0.2577<br>p_{FDR} = 0.001
**Central Executive Network (CEN)**	rho = 0.1464<br>p_{FDR} = 0.001	rho = 0.2614<br>p_{FDR} = 0.001
**Posterior Default Mode Network (pDMN)**	rho = 0.1165<br>p_{FDR} = 0.001	rho = 0.2142<br>p_{FDR} = 0.054
**Motor-sensory Network (MSN)**	rho = 0.1829<br>p_{FDR} = 0.001	rho = 0.2343<br>p_{FDR} = 0.001
**Visual Network 1 (VI)**	rho = 0.1381<br>p_{FDR} = 0.001	rho = 0.2537<br>p_{FDR} = 0.001
**Visual Network 2 (VII)**	rho = 0.1321<br>p_{FDR} = 0.054	rho = 0.2264<br>p_{FDR} = 0.001
**Visual Association Network (VAs)**	rho = 0.1419<br>p_{FDR} = 0.037	rho = 0.2348<br>p_{FDR} = 0.001
**Salience Network (SN)**	rho = 0.2010<br>p_{FDR} = 0.001	rho = 0.2597<br>p_{FDR} = 0.001
**Subcortical Network (SC)**	rho = 0.1654<br>p_{FDR} = 0.001	rho = 0.2094<br>p_{FDR} = 0.001
**Cerebellar Network (CBL)**	rho = 0.1522<br>p_{FDR} = 0.019	rho = 0.2275<br>p_{FDR} = 0.001

© 2025 Ben-Zion Z et al. *JAMA Network Open*.

eTable 4. Top Predicting Nodes

Degree centrality of the top predicting nodes at 1-month and 14-months post-trauma. Node numbers and names are defined by Shen et al. (2013)[24], ordered by their degree centrality (high to low). For visualization, see https://bioimagesuiteweb.github.io/webapp/connviewer.html?species=human.

Node number	Node name	Network assignment	Degree at 1-month	Degree at 14-months
146	left Prefrontal, dlPFC (dorsal) (BA9)	SAL	108	115
151	left Prefrontal, Pars Orbitalis of the Inferior Frontal Gyrus (BA47)	aDMN	99	76
183	left Parietal, Angular Gyrus (BA39)	aDMN	98	100
192	left Temporal, Medial Temporal Gyrus (BA21)	aDMN	94	76
7	right Prefrontal, Anterior PFC (BA10)	CEN	86	84
8	right Prefrontal, Anterior PFC (BA10)	CEN	84	80
139	left Prefrontal, Anterior PFC (BA10)	CEN	81	84
47	right Parietal, Supramarginal Gyrus (BA40)	CEN	77	69
12	right Prefrontal, Frontal Eye Fields (BA8)	aDMN	75	66
156	left Prefrontal, Broca-Operculum (BA44)	aDMN	75	0
188	left Temporal, Temporal Pole (BA38)	SMN	73	0
76	right Occipital, Secondary Visual (BA18)	VII	73	75
16	right Prefrontal, Pars Orbitalis of the Inferior Frontal Gyrus (BA47)	aDMN	71	0
10	right Prefrontal, dlPFC (dorsal)(BA9)	aDMN	69	80
17	right Prefrontal, Pars Orbitalis (BA47)	CEN	0	66
143	left Prefrontal, Anterior PFC (BA10)	CEN	0	66
59	right Temporal, Inferior Temporal Gyrus (BA20)	VAs	0	65

© 2025 Ben-Zion Z et al. *JAMA Network Open*.

eFigure 1. Unique Edges Predicting PTSD Symptoms at 1 Month vs 14 Months

Brain regions that were specific to the CAPS-5 total score predictions at each time point. The top part presents the degree centrality of the nodes that negatively predicted CAPS-5 scores only at 1-month or 14-month post-trauma (panels **A** and **B**, respectively). The bottom part shows the number of negatively predicting connections within and between canonical functional brain networks, unique to 1-month or 14-months symptom prediction (panels **C** and **D**, respectively).

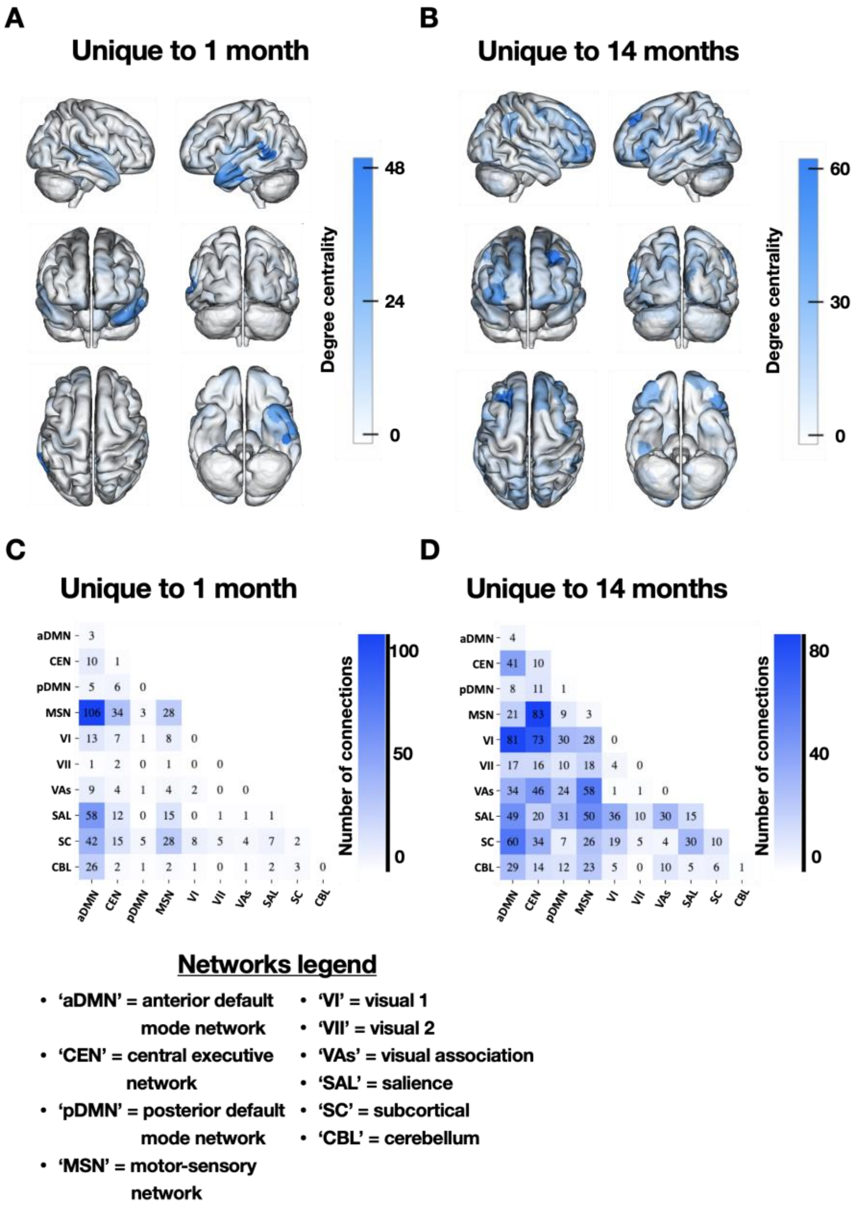

© 2025 Ben-Zion Z et al. *JAMA Network Open*.

eFigure 2. Shared Edges Predicting PTSD Symptoms at Both 1 Month and 14 Months

Brain regions that predicted CAPS-5 total scores at both time points. Panel **A** presents the degree of centrality of the nodes that negatively predicted CAPS-5 scores. Panel **B** shows the number of negatively predicting connections within and between canonical functional brain networks.

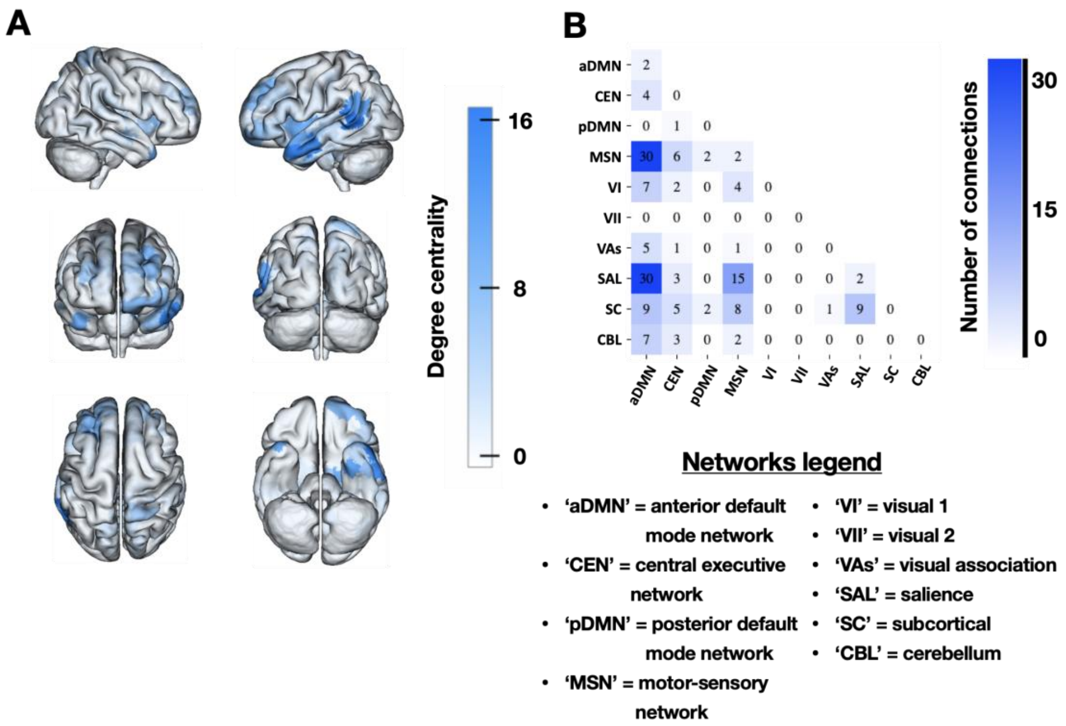

© 2025 Ben-Zion Z et al. *JAMA Network Open*.

eFigure 3. Brain Networks Contributing to Specific PTSD Symptom Clusters at 1 Month

Assignments of predictive edges at 1-month post-trauma for criterion B (intrusiveness), criterion C (avoidance), criterion D (negative mood and cognition), and criterion E (hyperarousal) (panels **A, B, C,** and **D**, respectively).

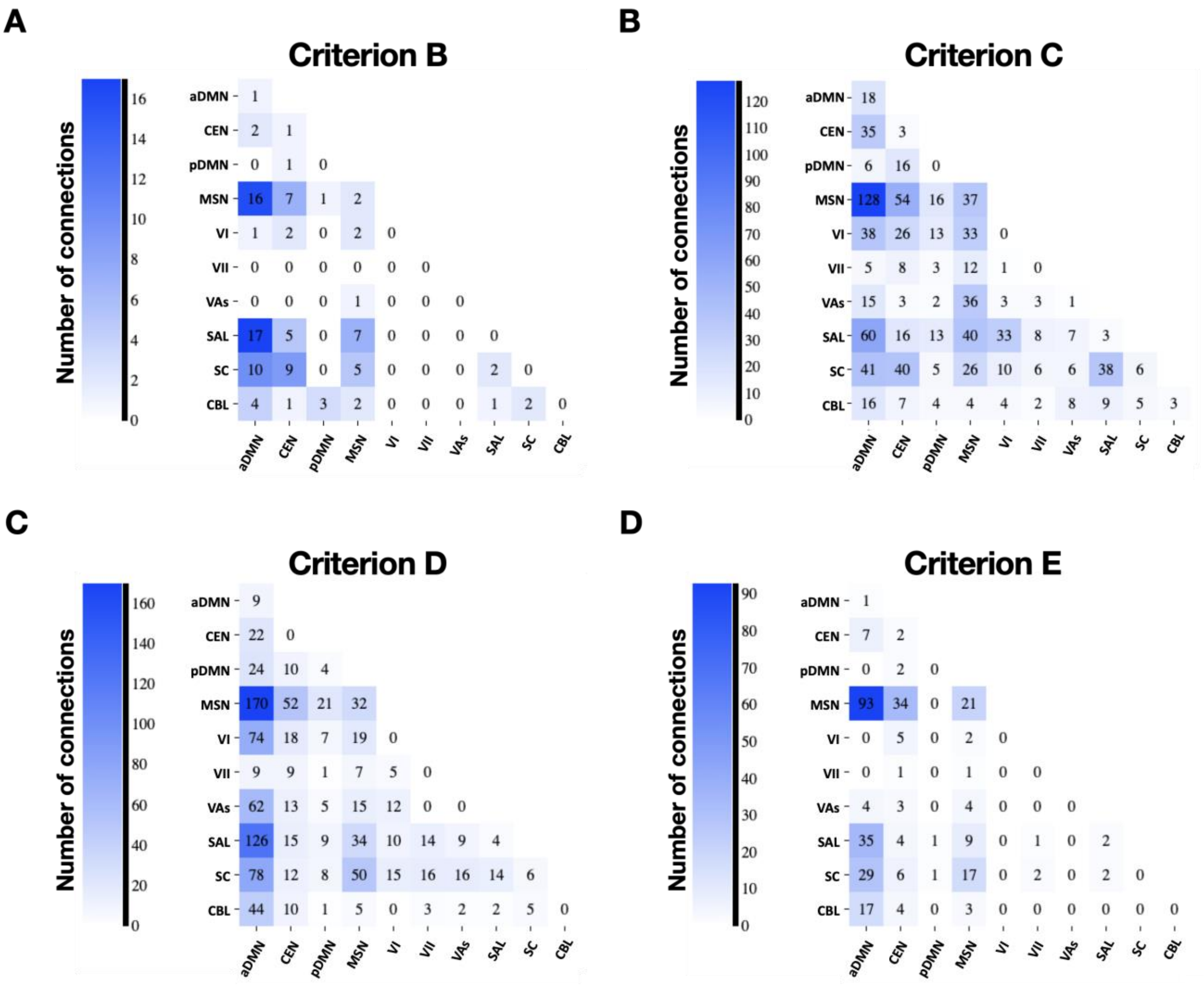

© 2025 Ben-Zion Z et al. *JAMA Network Open*.

eFigure 4. Brain Networks Contributing to Specific PTSD Symptom Clusters at 14 Months

Assignments of predictive edges at 14-months post-trauma for criterion B (intrusiveness), criterion C (avoidance), criterion D (negative mood and cognition), and criterion E (hyperarousal) (panels **A, B, C,** and **D**, respectively).

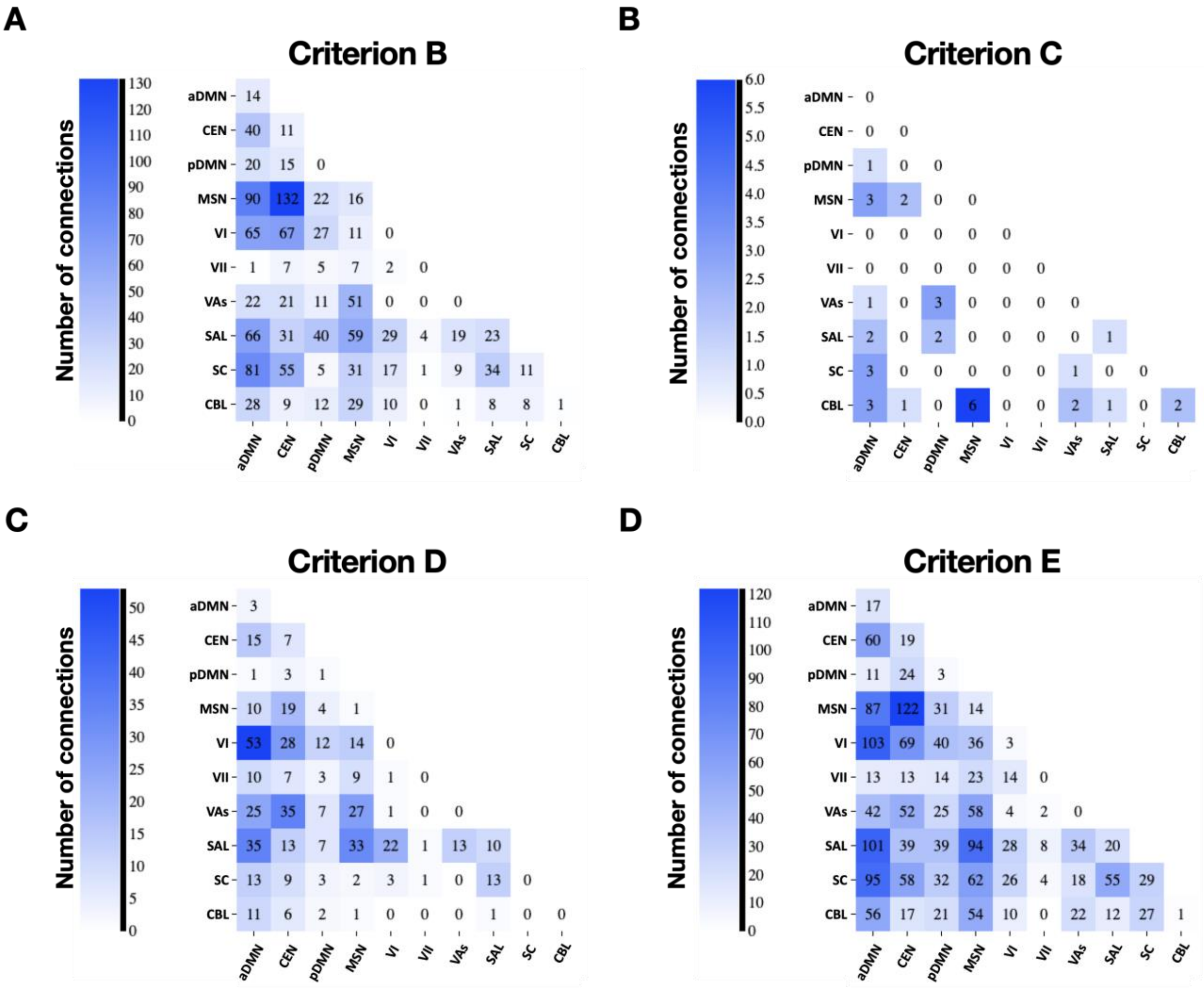

© 2025 Ben-Zion Z et al. *JAMA Network Open*.

eReferences

1. Hariri, A. R., Bookheimer, S. Y. & Mazziotta, J. C. Modulating emotional responses: effects of a neocortical network on the limbic system. *NeuroReport* **11**, 43–48 (2000).
2. Ben-Zion, Z. *et al.* Neural Responsivity to Reward Versus Punishment Shortly After Trauma Predicts Long-Term Development of Posttraumatic Stress Symptoms. *Biological Psychiatry: Cognitive Neuroscience and Neuroimaging* **7**, 150–161 (2022).
3. Kahn, I. *et al.* The role of the amygdala in signaling prospective outcome of choice. *Neuron* **33**, 983–94 (2002).
4. Admon, R. *et al.* Imbalanced neural responsivity to risk and reward indicates stress vulnerability in humans. *Cerebral Cortex* **23**, 28–35 (2013).
5. Assaf, M. *et al.* Brain activity dissociates mentalization from motivation during an interpersonal competitive game. *Brain Imaging and Behavior* **3**, 24–37 (2009).
6. Thaler, A. *et al.* Altered reward-related neural responses in non-manifesting carriers of the Parkinson disease related LRRK2 mutation. *Brain Imaging and Behavior* **13**, 1009–1020 (2019).
7. Hyatt, C. J. *et al.* Reward-related dorsal striatal activity differences between former and current cocaine dependent individuals during an interactive competitive game. *PLoS ONE* **7**, 1–15 (2012).
8. Gonen, T., Admon, R., Podlipsky, I. & Hendler, T. From animal model to human brain networking: Dynamic causal modeling of motivational systems. *Journal of Neuroscience* **32**, 7218–7224 (2012).
9. Gorgolewski, K. J. *et al.* The brain imaging data structure, a format for organizing and describing outputs of neuroimaging experiments. *Scientific Data* **3**, (2016).
10. Esteban, O. *et al.* MRIQC: Advancing the automatic prediction of image quality in MRI from unseen sites. *PLoS ONE* **12**, e0184661 (2017).
11. Esteban, O. *et al.* fMRIPrep: a robust preprocessing pipeline for functional MRI. *Nature Methods* **16**, 111–116 (2019).
12. Gorgolewski, K. *et al.* Nipype: A flexible, lightweight and extensible neuroimaging data processing framework in Python. *Frontiers in Neuroinformatics* **5**, (2011).
13. Tustison, N. J. *et al.* N4ITK: Improved N3 bias correction. *IEEE Transactions on Medical Imaging* **29**, 1310–1320 (2010).

© 2025 Ben-Zion Z et al. *JAMA Network Open*.

14. Fonov, V., Evans, A., McKinstry, R., Almli, C. & Collins, D. Unbiased nonlinear average age-appropriate brain templates from birth to adulthood. *NeuroImage* **47**, S102 (2009).

15. Zhang, Y., Brady, M. & Smith, S. Segmentation of brain MR images through a hidden Markov random field model and the expectation-maximization algorithm. *IEEE Transactions on Medical Imaging* **20**, 45–57 (2001).

16. Greve, D. N. & Fischl, B. Accurate and robust brain image alignment using boundary-based registration. *NeuroImage* **48**, 63–72 (2009).

17. Jenkinson, M., Bannister, P., Brady, M. & Smith, S. Improved optimization for the robust and accurate linear registration and motion correction of brain images. *NeuroImage* **17**, 825–841 (2002).

18. Behzadi, Y., Restom, K., Liau, J. & Liu, T. T. A component based noise correction method (CompCor) for BOLD and perfusion based fMRI. *NeuroImage* **37**, 90–101 (2007).

19. Lanczos, C. Evaluation of Noisy Data. *Journal of the Society for Industrial and Applied Mathematics Series B Numerical Analysis* **1**, 76–85 (1964).

20. Abraham, A. *et al.* Machine learning for neuroimaging with scikit-learn. *Frontiers in Neuroinformatics* **8**, 14 (2014).

21. Finn, E. S. *et al.* Can brain state be manipulated to emphasize individual differences in functional connectivity? *NeuroImage* **160**, 140–151 (2017).

22. Noble, S. *et al.* Influences on the Test–Retest Reliability of Functional Connectivity MRI and its Relationship with Behavioral Utility. *Cerebral Cortex* **27**, 5415–5429 (2017).

23. Greene, A. S., Gao, S., Scheinost, D. & Constable, R. T. Task-induced brain state manipulation improves prediction of individual traits. *Nat Commun* **9**, 2807 (2018).

24. Shen, X., Tokoglu, F., Papademetris, X. & Constable, R. T. Groupwise whole-brain parcellation from resting-state fMRI data for network node identification. *Neuroimage* **82**, 403–415 (2013).

© 2025 Ben-Zion Z et al. *JAMA Network Open*.
