# Supplementary material for: Connectome-Based Predictive Modeling of PTSD Development Among Recent Trauma Survivors
Source: JAMA Netw Open. 2025 Mar 10;8(3):e250331. doi: 10.1001/jamanetworkopen.2025.0331 (PMC11894499; doi:10.1001/jamanetworkopen.2025.0331)
Supplement: Supplement 2. — Data Sharing Statement [file jamanetwopen-e250331-s002.pdf]

Data Sharing Statement

Ben-Zion. Connectome-Based Predictive Modeling of PTSD Development Among Recent Trauma Survivors. *JAMA Netw Open*. Published March 10, 2025.
doi:10.1001/jamanetworkopen.2025.0331

Data
Data available: No

Additional Information
Explanation for why data not available: Python scripts for data cleaning and extracting time courses for the fMRI data are aviliable on GitHub (https://github.com/LevyDecisionNeuroLab/CPM_RS_TLV). MATLAB scripts for performing the main CPM analyses are available on GitHub (https://github.com/asimon445/PTSD_CPM). Analytical and visualization tools can be accessed via BioImage Suite Web (https://bioimagesuiteweb.github.io/webapp/index.html). Additional data may be obtained upon reasonable request to Dr. Ziv Ben-Zion (ziv.ben-zion@yale.edu), subject to approval by the study's principal investigators.
